# Musician Conserved Absolute Pitch Ability Despite a Right Parietal Subcortical Hemorrhage

**DOI:** 10.7759/cureus.34319

**Published:** 2023-01-28

**Authors:** Masahito Katsuki, Yoichi Higo, Kenta Kashiwagi, Shin Kawamura, Akihito Koh

**Affiliations:** 1 Department of Neurosurgery, Itoigawa General Hospital, Itoigawa, JPN; 2 Department of Radiological Technology, Itoigawa General Hospital, Itoigawa, JPN; 3 Department of Neurology, Itoigawa General Hospital, Itoigawa, JPN

**Keywords:** neural function, music, stroke, dual-stream models on auditory processing, absolute pitch (perfect pitch)

## Abstract

Absolute pitch (AP) can identify and designate the pitch chroma of a particular tone without using any external references. Unknown neurological mechanisms underlie it. We report the case of a 53-year-old AP musician who developed a right parietal hemorrhage but conserved AP ability. Our case had a lesion in the right parietal lobe that did not affect her AP ability. Our case further supports the hypothesis that the left cerebral hemisphere is important for AP ability.

## Introduction

The capacity to identify and characterize the pitch chroma of a particular tone without using reference tones is known as absolute pitch (AP) [1-4]. It is not known what the precise neuronal architecture of AP is. Less than one in 10,000 people might have AP [2]. As a result, much AP research has focused on healthy volunteers [2,5], while documentation of AP in stroke patients is limited [6,7]. Heschl’s gyrus in the temporal lobes and other cerebral cortices in the frontal and parietal lobes [8,9], including the inferior frontal gyrus, pre-supplementary motor area, dorsolateral prefrontal cortex, and intraparietal sulcus, have been reported as potential significant brain structures for AP [2,8,9]. The actual brain process underlying AP is unknown, though. The patient described here had a right parietal subcortical hemorrhage but maintained her AP, indicating that the right parietal lobe may not be connected to AP ability. Our study aids in the explanation of the AP neuronal mechanism.

## Case presentation

A 53-year-old right-handed AP pianist who had a moderate headache that had lasted three days as well as minor topographical agnosia, which made her feel lost while driving, walked into our emergency room. She began playing the piano when she was five years old and later earned a degree in piano performance from a music college. She was a moderate student in the college's music dictation class and had been conscious of her AP since elementary school. After graduating, she managed a music school and performed piano at some events. She was admitted with a blood pressure of 152/79 mmHg due to untreated hypertension. A right parietal subcortical hemorrhage of about 8 mL was detected by head computed tomography. Nicardipine was used as an antihypertensive medicine in conservative therapy.

**Figure 1:**
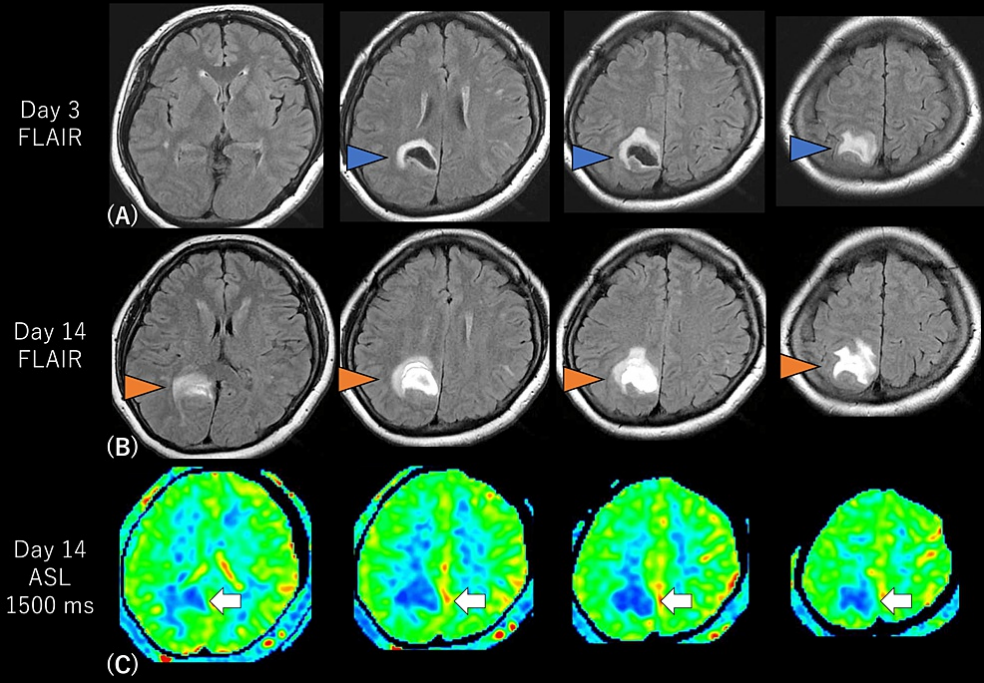
Radiological findings. The right parietal subcortical hematoma and surrounding edema showed on day 3 FLAIR images (blue arrowheads in A). The edematous lesion and hematoma remained on day 14 FLAIR images (orange arrowheads in B). The ASL images revealed hypoperfusion in the right parietal lobe (arrows in C). FLAIR: fluid-attenuated inversion recovery; ASL: arterial spin labeling.

Fluid-attenuated inversion recovery (FLAIR) images confirmed that the left Heschl’s gyrus, planum temporale, inferior frontal gyrus, pre-supplementary motor area, dorsolateral prefrontal cortex, and intraparietal sulcus, which are important to AP [4], were not injured. The hematoma is shown (blue arrowheads in Figure 1A). The edematous lesion slightly improved on day 14 (orange arrowheads in Figure 1B). The right parietal lobe, where the hematoma was located, showed hypoperfusion on the arterial spin labeling (ASL) images (arrows in Figure 1C).

On the next day (day 15), her AP was evaluated using a pitch-naming test in the rehabilitation room, using a Yamaha C1X Grand Piano (Yamaha Music Japan Co., Ltd., Hamamatsu, Japan) [2]. She was instructed to identify pitch classes (e.g., C, C#, and D) of 60 random piano tones covering five octaves. One investigator applied equal force to each key on the piano once. A random number table determined the order of the tones. The piano-tone noises lasted for roughly two seconds. The patient was given as much time as needed to react throughout this untimed test. Another investigator recorded her replies as she read aloud the note's name. Her precision was 88%. Nearly 80% of the responses were given within three seconds. We determined that her AP was conserved as a result.

Other measures of cognitive function did not show significant deficits, except for Kana pick-up test with 85% accuracy: The results of the Standard Language Test of Aphasia were 20/20, Communication Activities of Daily Living (ADL) test 136/136, Hasegawa Dementia Scale-Revised 30/30, Trail Making Test-A and -B 87 and 154 seconds, and Kohs block test intelligence quotient (IQ) 106. On day 27, she was discharged home with a modified Rankin Scale score of 0. She was back to working as a piano teacher soon, and there has been no particular interruption to her musical activities to date.

## Discussion

It is hypothesized that AP is related to the left-dominant hemispheric specialization of auditory cortical functions. AP possessors show left-dominant neural responses to musical stimuli, enhanced functional connectivity in the left superior temporal gyrus, enhanced superior longitudinal fasciculus, and greater left-dominant asymmetry of gray matter volume in perisylvian brain areas. Electroencephalography revealed T-complex’s left-right asymmetry in AP possessors compared to non-AP individuals [10]. There is a significant positive correlation between AP proficiency and fractional anisotropy values in the left white matter underlying the planum temporale in diffusion tensor imaging [1]. On the other hand, some studies reported the right hemisphere’s relationship to AP ability. The right auditory cortex’s volume was increased in AP individuals [11]. There might be a cerebral hemispheric asymmetry about the AP ability, but the exact difference remains unclear. Some studies reported that the parietal lobe is related to AP ability [8,9]. However, much is still unknown about the relationship between the AP and the parietal lobe.

The "dual-stream" model of auditory processing is proposed. A ventral or “what” stream is projecting from the primary auditory cortices along the anterior and middle temporal regions to the inferior frontal gyrus and parieto-occipital lobes via the middle longitudinal fasciculus (MdLF) and the inferior longitudinal fasciculus. The ventral stream processes spectrally complex sounds and facilitates auditory object recognition. A dorsal or “where” stream projects from primary auditory areas and planum temporale to the dorsolateral prefrontal cortex via the arcuate fiber and the superior longitudinal fasciculus. The dorsal stream is thought to support sensorimotor integration, motor commands, and verbal memory function. The ventral stream is believed to be important in AP because it is a specific type of auditory recognition where sounds are heard as discrete pitch categories and mapped onto meaningful labels. In contrast, the dorsal stream also provides verbal codes necessary for pitch labeling in AP, and the linkage between space and pitch is incorporated because we use the word “height.” It is unknown which neural stream in the AP's neural processes is crucial in this complex environment [5]. Both important fibers, MdLF and superior longitudinal fasciculus, project to the parietal lobes, so the parietal lobes may also relate to AP ability.

The parietal lobe is supposed to be related to AP ability. The left parietal lobe seems important for AP ability. The left MdLF projects more widely to the left parietal lobe in individuals with relative pitch compared to those without relative pitch [10]. Also, AP patients had left putaminal hemorrhage interfering with the left MdLF, and the ASL image showed hypoperfusion in the left parietal lobe, suggesting that the left MdLF and left parietal lobe are important for AP ability [7]. On the other hand, functional magnetic resonance imaging revealed activation in the right parietal cortex, reflecting pitch judgment and melodic transposition [9]. Previous reports were based on radiological studies, but the radiological investigation should be interpreted cautiously due to its low reproducibility. While hypotheses about the mechanism of AP are based on such ambiguous radiological investigation, our case had a lesion at the right parietal lobe that did not affect her AP ability. Our case further supports the hypothesis that the left cerebral hemisphere is important for AP ability. Further studies on AP ability among both healthy volunteers and those with neurological diseases are needed.

## Conclusions

AP musician developed a right parietal subcortical hemorrhage, but she maintained her AP. In this context, the right parietal lobe may not be related to AP. There are no standardized tests for AP, and radiological imaging should be interpreted cautiously. Moreover, our case had a lesion in the white matter but not the cortex. Therefore, the white matter may be more important than the cortex. However, our case is a rare AP study of a cerebral hemorrhage patient, not a healthy volunteer. Further case reports on AP in stroke patients are expected in the future to clarify the AP neural mechanism.
